# Dr. Collin's Case of Eleven Month's Pregnancy

**Published:** 1826-07

**Authors:** 


### ELEVEN MONTHS' PREGNANCY.*

Considerable interest is attached to this subject now, in consequence of a recent trial in high life, the issue of which is not yet determined, we believe. Nature is, no doubt, a most systematic legislator, but yet she does not always closely adhere to her own laws. Few things are more regular than the period of utero-gestation, both in the human and the brute creation; yet the peeress and the cow will sometimes go beyond the nine months, or bring forth their young short of that period. Without giving credit to the monstrous tales that have been recorded respecting extraordinary protractions of utero-gestation, we may fairly believe that ten, eleven, or perhaps twelve or fourteen months may elapse between conception and birth. The parliament of Paris, decided the

* Edinburgh Medical and Surgical Journal, April 1826. Dr. James Collins of Liverpool.

legitimacy of a child born eleven months after the departure of the father to another country. The university of Heidelburg allowed the legitimacy of a child born at the expiration of 13 months—which we think was a most extraordinary stretch of indulgence to the fair-sex. Moriceau, La Motte, and others, have related cases in their own practice, in which pregnancy continued 12 or 13 months. The *former* states that there are no fixed limits for utero-gestation—that it is influenced by many circumstances over which we have no control. The *latter*, after citing many remarkable cases that fell under his own observation, says, it is natural to imagine that the child who has taken more nourishment, may grow more in nine months than another in the same time; and asks why should not the fœtus that has not acquired the same degree of development, retain the place destined for this purpose, until it has attained the necessary degree of perfection? But we shall cease speculating, and proceed to facts.

The subject of Dr. Collins' case, was a woman about 24 years of age, and the mother of several children. She engaged our author to attend her, being then, as she calculated, in the eighth month of pregnancy. The fundus uteri was as high as the epigastric region, and gave to the abdomen the form and appearance of full eight months' pregnancy. Our author, however, not being satisfied with numerical information, proceeded to what he considers a much more certain criterion.

"In examining *per vaginam*, I found the neck or cervix of the uterus remarkably high, scarcely tangible, and with difficulty distinguished from the body of the uterus, as it presented little or no prolongation. Availing myself of my position, I placed the left hand on the abdomen, and, giving a gentle jerk to the *os tincæ* with the index finger of the other, the fœtus bounded from the touch, and fell again on the finger, exciting the sensation which the French call *ballotement*, and that degree of weight which a fœtus of eight months, it is supposed, could alone produce. Thus I ascertained the stage of the pregnancy; as the indications I have just detailed, according to the experience and observation of the most eminent in the profession, are sufficiently characteristic of it, and constitute the most conclusive means we possess to determine it with accuracy." 246.

We confess that we have less confidence in this process of tilting and balancing a fœtus on the point of the finger, for the purpose of ascertaining its age, than our author seems to have; and, for the reason given by La Motte above, viz. the different degrees of development in different fœtuses at the same period after conception. Be this as it may, the end of the ninth month approached—the uterus gradually sunk in the abdomen—and the patient began to feel all the sensations that usually accompany that state. Our author was again induced to make examination per vaginam, and found the head of the fœtus much lower down, and more solid to the touch; the os tincæ was open, and every thing announced speedy labour, the precursory symptoms, in fact, being already felt. Soon after the expiration of the ninth month she appeared to have labour-pains; but they went off, time after time, and she was thus harrassed for three or four weeks, without things coming to a crisis. Our author then began to grow uneasy, and to suspect that he had made a false calculation. He resumed his experiments and examinations, but found the parts just as they were at the expiration of the nine months. He, therefore, diminished the frequency of his visits, and two months had nearly elapsed since the expiration of the ordinary time of pregnancy, without any more probability of accouchement. At length the symptoms which manifested themselves at the close of the ninth month returned, and after a very short labour the woman was delivered of a fine active boy. There was only one thing remarkable in the parturition. The membranes did not give way until the head was almost at the extremity of the passage. The moment they broke, the infant screamed, and excited no small amazement in the mother and byestanders.

Dr. Collins informs us that this poor woman's husband lost his situation about the third month of her pregnancy, and, in hopes of procuring another, he continued in Liverpool until his wife's pregnancy was so far advanced as to prevent her accompanying him to Ireland where he might have got another. He at length, when the pregnancy was so far over-run, was obliged to leave her in the greatest distress. We draw a conclusion different from our author on this point. We think it was natural for the wife to represent herself as farther gone with child than she really was, in order to induce the husband not to abandon her in such a situation. Still the case is remarkable, and offers, we believe, an instance of protracted utero-gestation.

